# Supplementary material for: Chilling- and dark-regulated photoprotection in Miscanthus, an economically important C4 grass
Source: Commun Biol. 2024 Dec 19;7:1660. doi: 10.1038/s42003-024-07320-0 (PMC11659412; doi:10.1038/s42003-024-07320-0)
Supplement: Supplementary file 2 — Supplemental Material [file 42003_2024_7320_MOESM2_ESM.pdf]

Chilling- and dark-regulated photoprotection in *Miscanthus*, an economically important C_4 grass

Jared Haupt, Katarzyna Glowacka

Supplementary material

Fig. S1. Weather data for UIUC and UNL field trials during four growing seasons.

Fig. S2. Chilling-induced differences in NPQ kinetics in *Miscanthus* grown at UIUC field.

Fig. S3. Chilling-induced differences in NPQ kinetics in *Miscanthus* grown at UNL field.

Fig. S4. Chilling- and light-induced changes in xanthophyll cycle pigments in two additional *Miscanthus* accessions.

Fig. S5. Chilling-induced differences in photosynthesis related pigments estimated from hyperspectral indexes in *Miscanthus* grown at UNL field.

Fig. S6. Differences in NPQ after norflurazon or nigericin infiltration in three *Miscanthus* accessions.

Fig. S7. Chilling- and light-induced changes in ascorbate and dehydroascorbate contents for two additional *Miscanthus* accessions.

Fig. S8. Transcript abundance of genes associated with NPQ regulation for three *Miscanthus* accessions in five combinations of chilling and high light stress.

Table S1 Primer sequences used in RT-qPCR.

Note 1: MATLAB code to fit NPQ induction to hyperbolic equation.

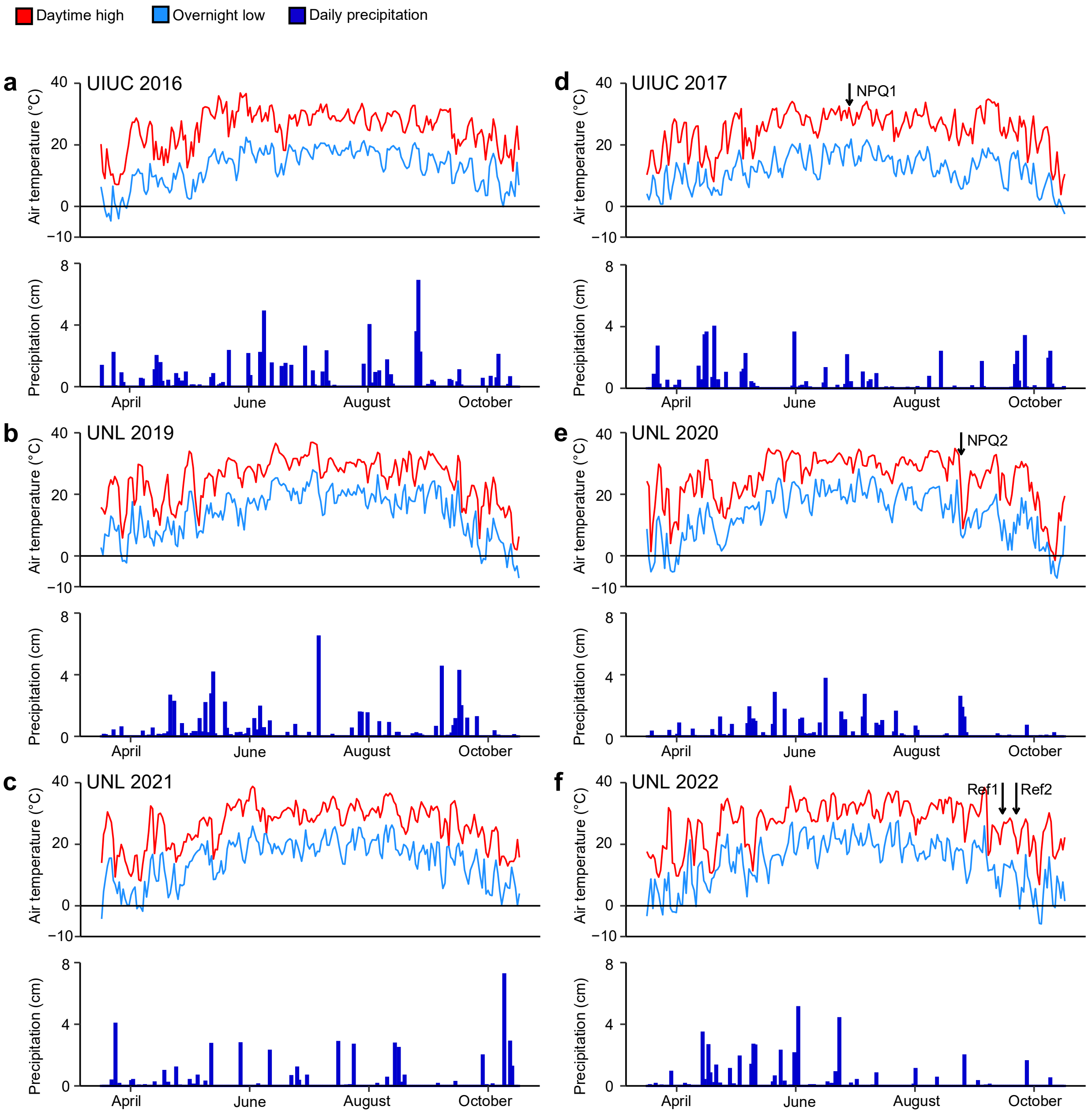

Fig. S1. Weather data for University of Illinois Urbana-Champaign (UIUC) and University of Nebraska-Lincoln (UNL) field trials during four growing seasons. Daytime high temperature (red), overnight low temperature (light blue) and 24-hour precipitation (dark blue) as recorded by weather stations (UIUC 40.067 N, 88.198 W; UNL, 40.828 N, 96.657 W). Arrows indicate the dates when samples were collected and are indicated by type of sample. Leaf discs were collected for NPQ kinetics from the UIUC experimental field on 2017-07-13 (NPQ1). Leaf discs were collected for NPQ kinetics from the UNL experimental field on 2020-09-08 (NPQ2). Leaf reflectance indices were measured from plants growing in the UNL field after warm overnight temperatures on 2022-09-29 (Ref1) or chilling overnight temperatures on 2022-10-06 (Ref2). NPQ1 corresponds to data shown in Fig. 1 and Fig. S2. NPQ2 corresponds to Fig. S3. Ref1 and Ref2 correspond to Fig. S5.

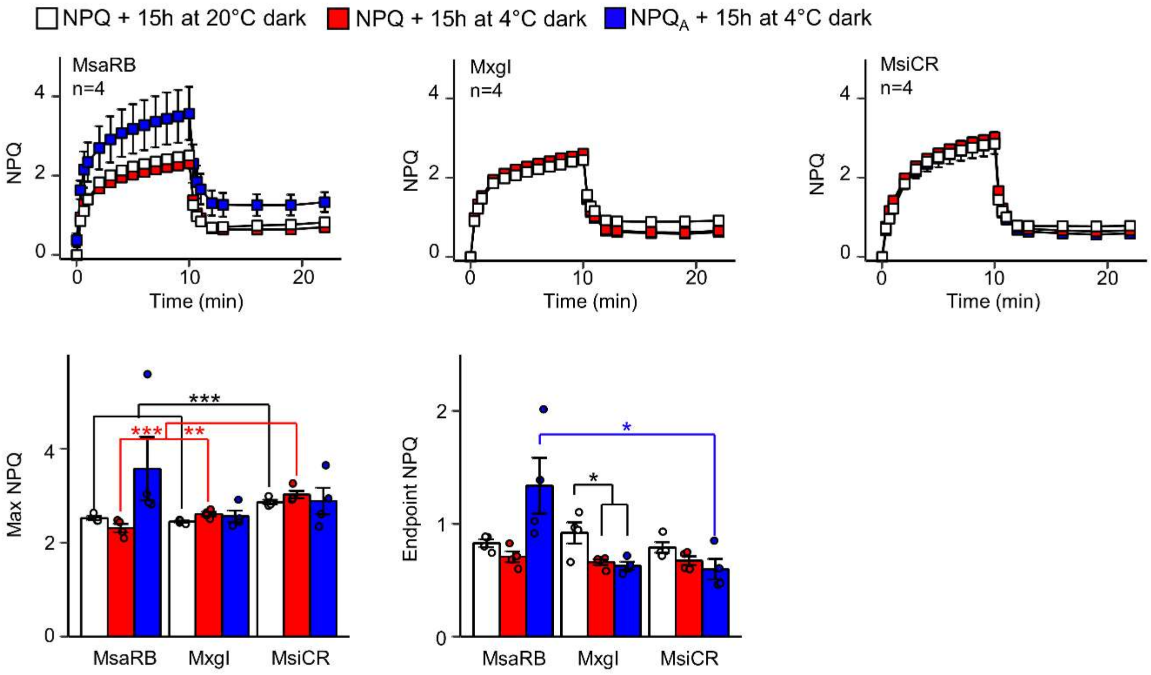

Fig. S2. Chilling-induced differences in NPQ kinetics from three *Miscanthus* accessions grown at the University of Illinois Urbana-Champaign experimental field (40.067 N, 88.198 W). NPQ kinetic measured and adjusted to initial quenching (NPQ_A) during 10 minutes of induction (light) and 10 minutes relaxations (dark) are shown. NPQmax and NPQend refer to the highest value of NPQ during the induction and NPQ in the last point in dark, respectively. The youngest fully expanded leaves were collected from 2-year-old, field-grown *M. sacchariflorus* Robustus-Blumel (MsaRB, high chilling tolerance), *M. ×giganteus* Illinois (MxgI; moderate chilling tolerance) and *M. sinensis* var. condensatus 'Cosmo Revert' (MsiCR). Values are means ± standard error from 4 biological replicates. In the line graphs, blue data points are hidden behind red points for MxgI and MsiCR accessions. In bar graphs significance was determined compared to MsiCR or compared to the warm treatment by Dunnett's post-hoc test. *$p < 0.05$; **$p < 0.01$; ***$p < 0.001$. Curves correspond to data shown in Fig. 1c and d. The weather data corresponding to measurements are shown in Fig. S1.

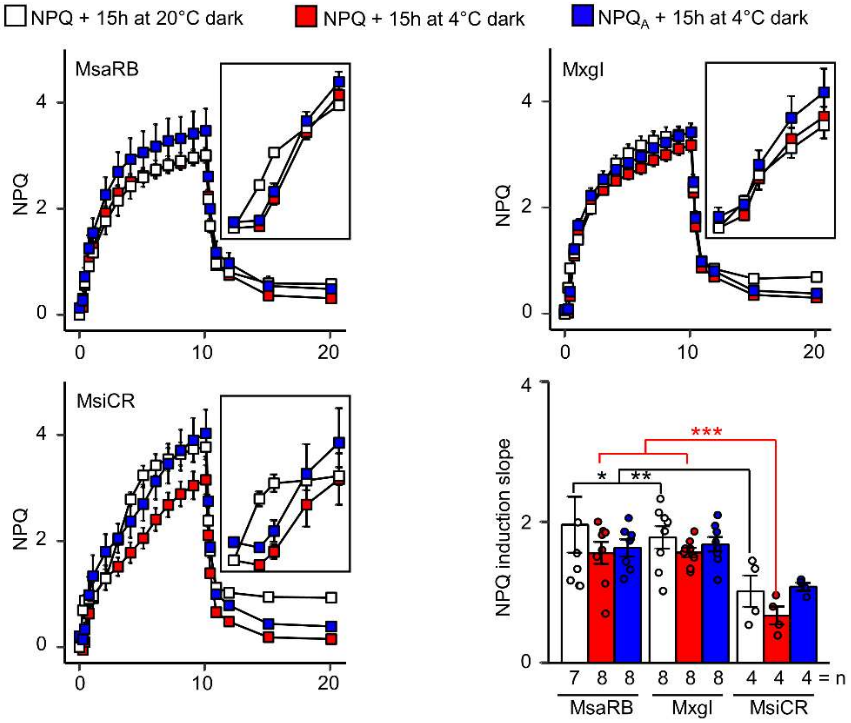

Fig. S3. Differences in NPQ kinetics following chilling overnight treatment in three *Miscanthus* accessions grown at the University of Nebraska-Lincoln experimental field (40.829 N, 96.657 W). Kinetic measurements of NPQ induction and relaxation at 20°C or 4°C during the 22-min duration of the assay. NPQ induction at 4°C adjusted to initial quenching (NPQ_A) is also shown. The first minute of NPQ induction is shown in greater detail in the inset. The slope of NPQ induction was calculated from a hyperbolic fit to NPQ and NPQ_A curves. The youngest fully expanded leaves were sampled from *M. sacchariflorus* Robustus-Blumel (MsaRB, high chilling tolerance), *M.* ×*giganteus* Illinois (MxgI; moderate chilling tolerance) and *M. sinensis* var. condensatus 'Cosmo Revert' (MsiCR). Values are means ± standard error (n = 4–8 biological replicates). Asterisks in panel indicate significance determined compared to MsiCR by Dunnett's post-hoc test. *$p < 0.05$; **$p < 0.01$; ***$p < 0.001$. The weather data corresponding to measurements are shown in Fig. S1.

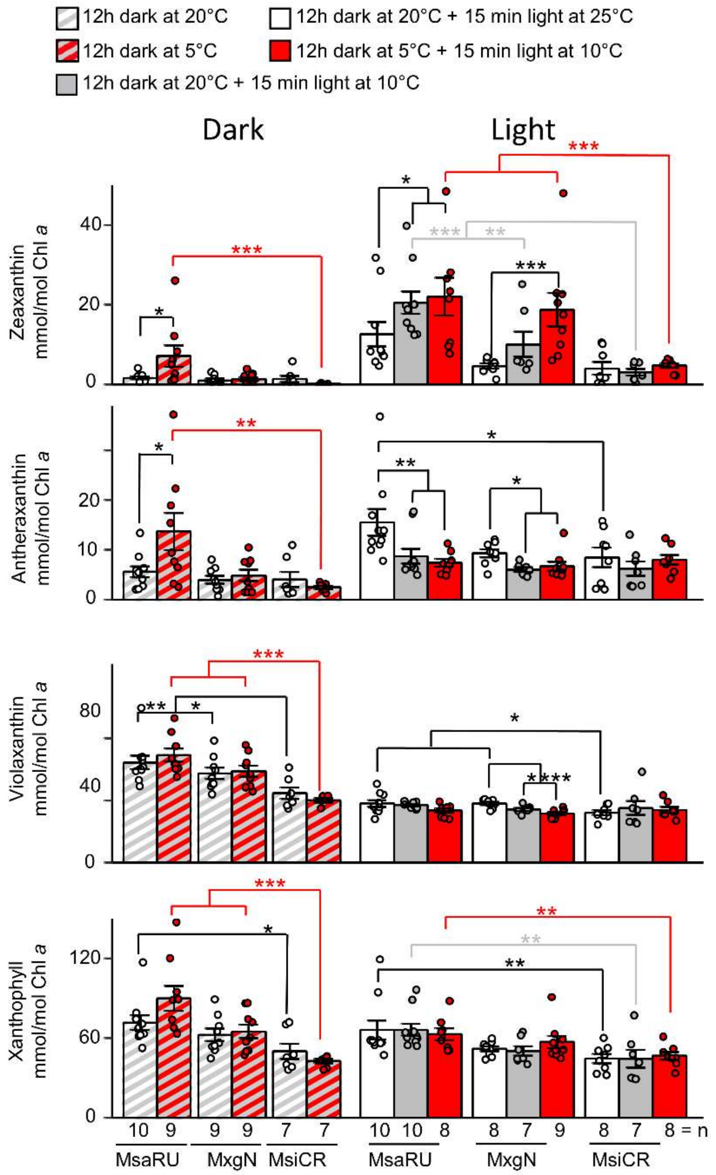

Fig. S4. Chilling- and light-induced changes in the contents of xanthophyll cycle pigments in two additional *Miscanthus* accessions. Contents of xanthophyll cycle pigments and xanthophyll de-epoxidation state in leaf samples from additional *Miscanthus* accessions with high chilling tolerance (MsaRU; *M. sacchariflorus* RU2012-114) or moderate chilling tolerance (MxgN; *M. ×giganteus* Nagara). For comparison, the data shown in Fig. 2 for MsiCR (low chilling tolerance) are replotted. Plants grown for 6 weeks at 25°C/20°C (day/night) were treated with one of five combinations of temperature and light treatments prior to sampling: 20°C in darkness; 5°C in darkness; 20°C in darkness + 25°C under 900 µmol m^{-2} s^{-1}; 20°C in darkness + 10°C under 900 µmol m^{-2} s^{-1}; 5°C in darkness + 10°C under 900 µmol m^{-2} s^{-1}. The youngest fully expanded leaves were collected for quantification of xanthophylls. Values are means ± standard error from 7–10 biological replicates. Significance was determined across accessions compared to MsiCR or within each accession compared to warm treatment by Conover-Iman test. *$p < 0.05$; **$p < 0.01$; ***$p < 0.001$.

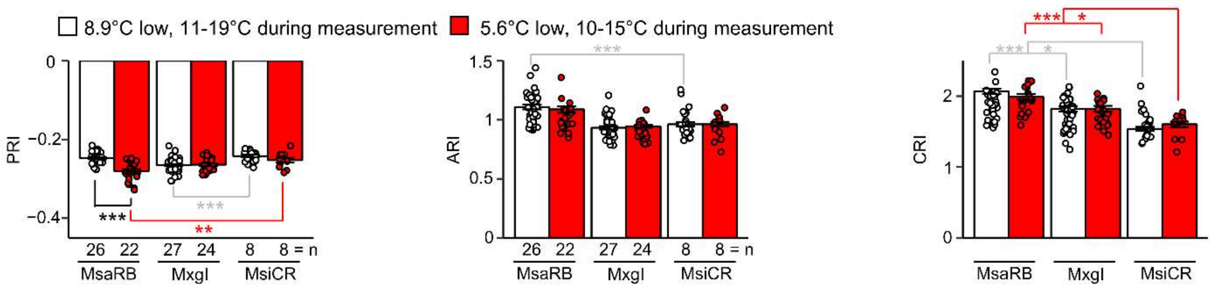

Fig. S5 Chilling-induced differences in photosynthesis related pigments estimated from hyperspectral indexes in field-grown plants of three *Miscanthus* accessions differing in chilling tolerance. Photochemical reflectance index (PRI), anthocyanin reflectance index (ARI) and carotenoid reflectance index (CRI) during warm or chilling morning for plants grown in the field in Lincoln, NE, USA (40.829 N, 96.657 W). All numbers represent means ± standard error for n = individual measurements defined as unique leaf and measurement round, from 5 to 16 biological replicates. significance was determined by Dunnett's post-hoc test within accessions with warm treatment as control (black) or within treatments with MsiCR as control (corresponding color). *$p < 0.05$; **$p < 0.01$; ***$p < 0.001$. MsaRB, *M. sacchariflorus* Robustus-Blumel (high chilling tolerance); MxgI, *M. ×giganteus* Illinois (moderate tolerance); MsiCR, *M. sinensis* var. condensatus 'Cosmo Revert' (low tolerance). The weather data corresponding to measurements are shown in Fig. S1.

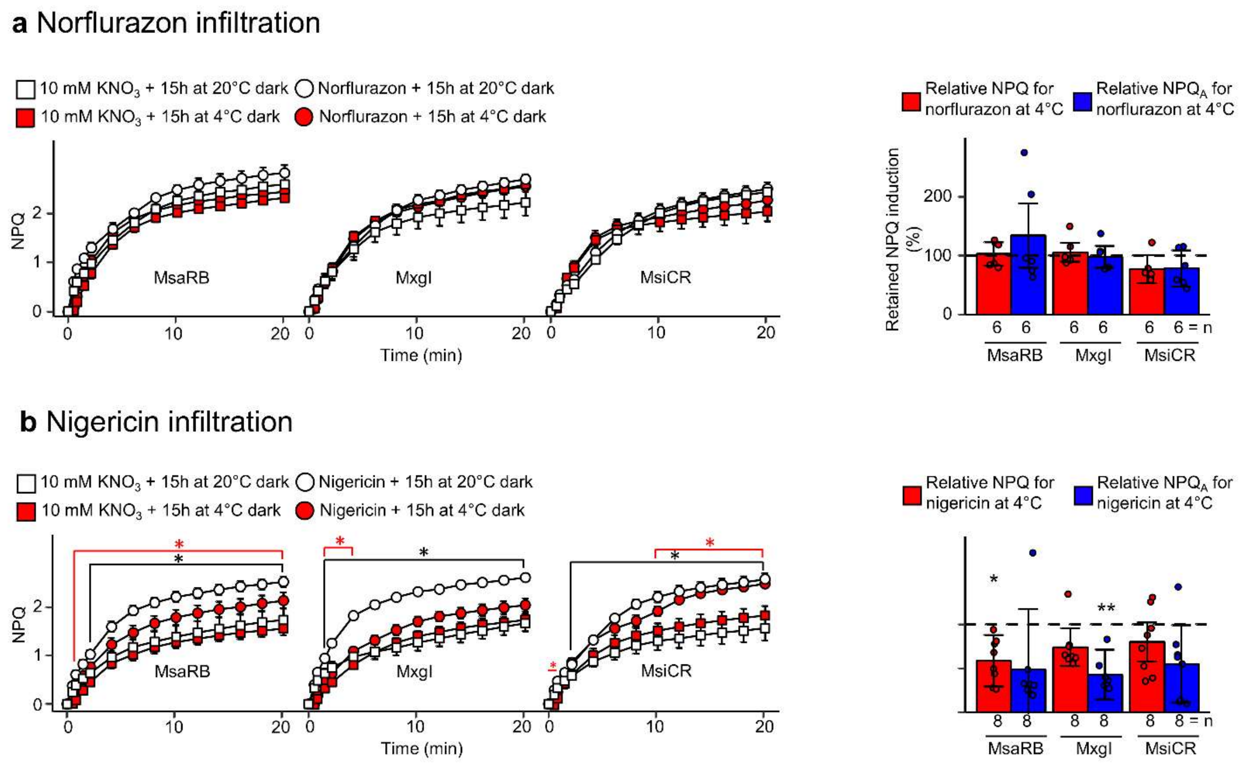

Fig. S6. Differences in NPQ for three *Miscanthus* accessions in response to norflurazon or nigericin infiltration and different night temperatures. (a) Effect of norflurazon, an inhibitor of *de novo* formation of zeaxanthin**,** and **(b)** the ionophore nigericin, which eliminates the pH gradient across the thylakoid membrane, under warm (20°C) or chilling (4ºC) night on NPQ induction the following morning. Leaf discs of plants grown in growth chambers at 25°C/20°C (day/night) were infiltrated with the indicated chemical or with the solvent as control and incubated overnight at 4°C or 20°C, followed by a NPQ kinetics assay during the first 10 min in the light. The relative rate of NPQ induction was estimated from the measured curve or curve adjusted to initial quenching (NPQ_A) and normalized to the corresponding solvent treatment (the last subpanels in each panel). The youngest fully expanded leaves were sampled for infiltration from *M. sacchariflorus* Robustus-Blumel (MsaRB, high chilling tolerance), *M.* ×*giganteus* Illinois (MxgI; moderate chilling tolerance) and *M. sinensis* var. condensatus 'Cosmo Revert' (MsiCR). For the time courses, significance was determined by a Student's t-test between chemical treatment and solvent at each time point and temperature. A Student's t-test was performed to compare NPQ induction rate for chemical treatment to solvent infiltration at 4°C. Values are means ± standard error from n = 8 biological replicates. *$p < 0.05$; **$p < 0.01$; ***$p < 0.001$.

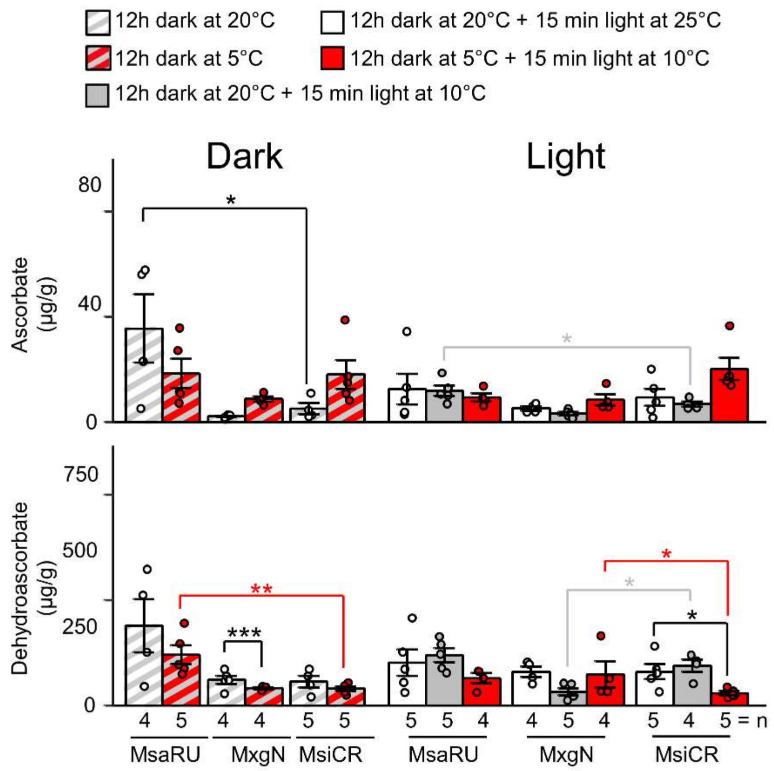

Fig. S7. Chilling- and light-induced changes in ascorbate and dehydroascorbate contents for two additional *Miscanthus* accessions. Contents for ascorbate and dehydroascorbate in leaf samples from *Miscanthus* accessions with high chilling tolerance (MsaRU; *M. sacchariflorus* RU2012-114) or moderate chilling tolerance (MxgN; *M. ×giganteus* Nagara). For comparison, the data shown in Fig. 5 for MsiCR (low chilling tolerance) are replotted. Plants grown for 6 weeks at 25°C/20°C (day/night) were treated with one of five combinations of temperature and light treatment prior to sampling: 20°C in darkness; 5°C in darkness; 20°C in darkness + 25°C under 900 µmol m^{-2} s^{-1}; 20°C in darkness + 10°C under 900 µmol m^{-2} s^{-1}; 5°C in darkness + 10°C under 900 µmol m^{-2} s^{-1}. The youngest fully expanded leaves were sampled for quantification of metabolites. Values are means ± standard error from 4–5 biological replicates. Significance was determined across accessions compared to MsiCR and within each accession compared to warm treatment by Dunnett's post-hoc test. *$p < 0.05$; **$p < 0.01$; ***$p < 0.001$.

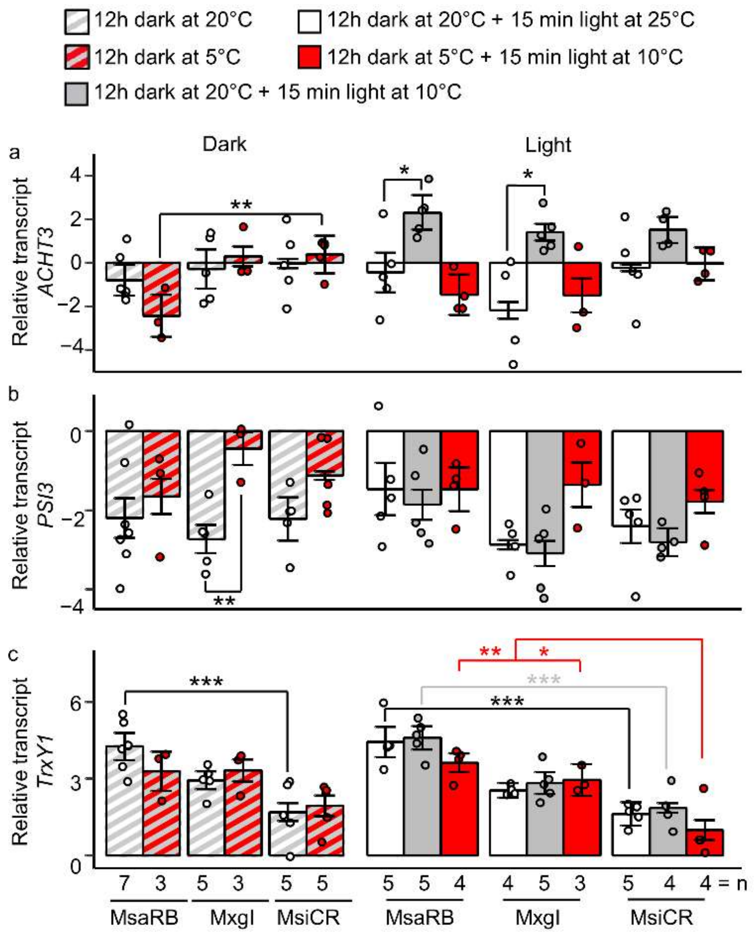

Fig. S8. Transcript abundance of three genes associated with NPQ regulation in leaves of three *Miscanthus* accessions subjected to five combinations of chilling and high light stress. Relative transcript levels for **(a)** *Atypical Cys His Rich Thioredoxin* (*ACHT3*) **(b)** *Phytosulfokine Simulator 3* (*Psi3*) and **(c)** *Thioredoxin Y1* (*TrxY1*), in *M. sacchariflorus* Robustus-Blumel (MsaRB; high chilling tolerance), *M. ×giganteus* Illinois (MxgI; moderate chilling tolerance) and *M. sinensis* var. condensatus 'Cosmo Revert' (MsiCR; low chilling tolerance). Plants grown for 6 weeks at 25°C/20°C (day/night) were exposed to one of five combinations of temperature and light treatments prior to sampling: 20°C in darkness; 5°C in darkness; 20°C in darkness + 25°C under 900 µmol m^{-2} s^{-1}; 20°C in darkness + 10°C under 900 µmol m^{-2} s^{-1}; 5°C in darkness + 10°C under 900 µmol m^{-2} s^{-1}. Transcript levels were normalized to the mean of *Ubiquitin* and *Elongation factor 1α* transcript levels. Values are means ± standard error from 3–7 biological replicates. Significant differences were determined by Dunnett's post-hoc test across accessions compared to MsiCR or within each accession compared to warm treatment. *$p < 0.05$; **$p < 0.01$; ***$p < 0.001$.

Table S1. Primer sequences used in RT-qPCR.

Target	Forward (5'-3')	Reverse (5'-3')
EF	ACCCCTGCTTGATGCTCTT	AACCGGGACAGTTCCAATAC
Ubiquitin	TCTACCCTCCTCCACCTTCTCTT	CTCAACCTCCAGTGTGATTGT
PsbS	CATCATCGGCGAGATCATCA	CGAAGAAGAAGAGGACGTTGA
VDE	TGTACTGCAAGCGTCAGATATT	AGAAGGGACTACCAGAACTACA
ZEP	CCCAGTCACAAGGGTCATTAG	TCGACTACATGGCTTCCATTC
ACHT3	GCTCTGACTATTGCGTCTGTAT	TTCTCGCTGCATAGAGGATTG
DHAR	CCCAGAAGGTAAAGTGCCTATC	GGTTCAGGGTACTTCTCCTCTA
PSI3	TCCAAGAAGGCAAAGGAAGAG	CGAAGGGAATCGGTGGATTT
TrxY1	GCAGTCACGAAGTAGCACATA	CGCTTGCCAGCAATTTACC

Note 1: MATLAB code to fit NPQ induction to hyperbolic equation.

```
AllDataNPQ = csvread('AllDataNPQ.csv');

%%Initialize variables
AllFitParms1=zeros(numel(AllDataNPQ(:,1)),2);
AllGof1 = AllFitParms1(:,1);
%% set up induction fits
Time = [0,0.333333333,0.666666667,1,2,3,4,5,6,7,8,9,10];
fitOpt1 = fitoptions('Method', 'NonLinearLeastSquares', 'Lower',[0 0 ], 'Upper', [5 100 ], 'StartPoint',
[0.5 3 ]);

ft1=fittype('(x*a+b-( (x*a+b).^2-4*.5*x*a*b ).^0.5)./(2*.5)','options',fitOpt1);
% where "a" is rate of NPQ induction, "b" is asymptote of NPQ induction

h=waitbar(0,'fitting NPQ induction parameters...');

for i = 1:numel(AllDataNPQ(:,1))
% fit induction by leaf disc:


    [fp1,gof1]=fit(Time(1:13)',AllDataNPQ(i,1:13)',ft1);
    AllFitParms1(i,:) = coeffvalues(fp1);
    AllGof1(i) = gof1.adjrsquare;


    plot(Time(1:13)',AllDataNPQ(i,1:13)','o')
    hold off

    waitbar(i/numel(AllDataNPQ(:,1)),h);
end

%% write outputfiles
csvwrite('AllFitParms.csv',[AllFitParms1,AllGof1]);
```
