# Supplementary material for: Chilling- and dark-regulated photoprotection in Miscanthus, an economically important C4 grass
Source: Commun Biol. 2024 Dec 19;7:1660. doi: 10.1038/s42003-024-07320-0 (PMC11659412; doi:10.1038/s42003-024-07320-0)
Supplement: Supplementary file 3 — Description of Additional Supplementary File [file 42003_2024_7320_MOESM3_ESM.pdf]

Description Of Additional Supplementary File

File name: Supplementary Data

Description: The source data behind the graphs in the main paper and graphs in supplementary Information file.
