# Supplementary material for: Chilling- and dark-regulated photoprotection in Miscanthus, an economically important C4 grass
Source: Commun Biol. 2024 Dec 19;7:1660. doi: 10.1038/s42003-024-07320-0 (PMC11659412; doi:10.1038/s42003-024-07320-0)
Supplement: Supplementary file 5 — Reporting Summary [file 42003_2024_7320_MOESM5_ESM.pdf]

nature portfolio

Corresponding author(s): Katarzyna Glowacka

Last updated by author(s): Oct 31, 2024

Reporting Summary

Nature Portfolio wishes to improve the reproducibility of the work that we publish. This form provides structure for consistency and transparency in reporting. For further information on Nature Portfolio policies, see our Editorial Policies and the Editorial Policy Checklist.

Statistics

For all statistical analyses, confirm that the following items are present in the figure legend, table legend, main text, or Methods section.

n/a	Confirmed	
☐	☒	The exact sample size (*n*) for each experimental group/condition, given as a discrete number and unit of measurement
☐	☒	A statement on whether measurements were taken from distinct samples or whether the same sample was measured repeatedly
☐	☒	The statistical test(s) used AND whether they are one- or two-sided<br>*Only common tests should be described solely by name; describe more complex techniques in the Methods section.*
☐	☒	A description of all covariates tested
☐	☒	A description of any assumptions or corrections, such as tests of normality and adjustment for multiple comparisons
☐	☒	A full description of the statistical parameters including central tendency (e.g. means) or other basic estimates (e.g. regression coefficient) AND variation (e.g. standard deviation) or associated estimates of uncertainty (e.g. confidence intervals)
☐	☒	For null hypothesis testing, the test statistic (e.g. *F*, *t*, *r*) with confidence intervals, effect sizes, degrees of freedom and *P* value noted<br>*Give P values as exact values whenever suitable.*
☒	☐	For Bayesian analysis, information on the choice of priors and Markov chain Monte Carlo settings
☒	☐	For hierarchical and complex designs, identification of the appropriate level for tests and full reporting of outcomes
☒	☐	Estimates of effect sizes (e.g. Cohen's *d*, Pearson's *r*), indicating how they were calculated

Our web collection on statistics for biologists contains articles on many of the points above.

Software and code

Policy information about availability of computer code

Data collection	MATLAB version R2019b; Multiquant 3.0 (Sciex)
Data analysis	R version 4.1.2 using the package DescTools 0.99.45 and PMCMRplus 1.9.6.

For manuscripts utilizing custom algorithms or software that are central to the research but not yet described in published literature, software must be made available to editors and reviewers. We strongly encourage code deposition in a community repository (e.g. GitHub). See the Nature Portfolio guidelines for submitting code & software for further information.

Data

Policy information about availability of data

All manuscripts must include a data availability statement. This statement should provide the following information, where applicable:

- Accession codes, unique identifiers, or web links for publicly available datasets
- A description of any restrictions on data availability
- For clinical datasets or third party data, please ensure that the statement adheres to our policy

All additional data that support the conclusions from this manuscript are available in the Supplementary Information. Source data for the main and supplementary figures are supplied as Excel file.

Research involving human participants, their data, or biological material

Policy information about studies with human participants or human data. See also policy information about sex, gender (identity/presentation), and sexual orientation and race, ethnicity and racism.

Reporting on sex and gender	N/A
Reporting on race, ethnicity, or other socially relevant groupings	N/A
Population characteristics	N/A
Recruitment	N/A
Ethics oversight	N/A

Note that full information on the approval of the study protocol must also be provided in the manuscript.

Field-specific reporting

Please select the one below that is the best fit for your research. If you are not sure, read the appropriate sections before making your selection.

☐ Life sciences ☐ Behavioural & social sciences ☒ Ecological, evolutionary & environmental sciences

For a reference copy of the document with all sections, see nature.com/documents/nr-reporting-summary-flat.pdf

Ecological, evolutionary & environmental sciences study design

All studies must disclose on these points even when the disclosure is negative.

Study description	We tested the effect of chilling in light and dark on multiple quantitative data including physiological parameters, metabolomic compounds and pigments. The design structure of experiments was factorial where genotype and treatment were considered. Experimental units were biological replicates. The exception was hyperspectral indices measured in the field for which experimental units were individual measurements defined as unique leaf and measurement round. In the case of gene expression, we also used two technical replicates for each biological replicate.
Research sample	The research samples were of leaves of three (in a few experiments five) Miscanthus genotypes belonging to three different species that are characterized by contrasting chilling tolerance (M. sinensis, M. sacchariflorus and M. x giganteus). In all experiments, measurements and leaf samples were collected from the center third of the youngest fully expanded leaf, avoiding the midrib.
Sampling strategy	In preliminary data collection, the number of four biological replicates showed after the chilling treatment already a significant difference in the main physiological trait which we studied. Therefore, we used at least four biological replicates in all our experiments. In a few cases, the outlier removal led to three biological replicates in the results shown in the figures. In multiple experiments, we have more than four biological replicates (up to 10). For the hyperspectral indices measured in the field for which experimental units were individual measurements defined as unique leaf and measurement round the number of experimental units was between 8 and 27.
Data collection	The instrument manuals were used to adopt the protocols for data collections. The first author was responsible for data records or the facility members who run the analyses (HPLC and LC-MS). After using the unique software for specific instrumentations data were archived as CVS or Excel file for further analyses.
Timing and spatial scale	For the growth chamber sampling of 15 minutes in the light the stacking of plants was applied, and plants were one by one moved from dark to light to undergo exactly 15 minutes of illumination priory to sampling. Dark samples were collected 30 min+/- 10 min before the end of the dark period. For using the natural chilling events in the field, the data were collected in the chilling morning after chilling night or warm morning after warm night (control). For NPQ kinetics leaf disks were collected at the end of the day (6 pm +/- 30 min), dark-adapted over-night and measured next morning. None of the data collection was longer than one day.
Data exclusions	If the statistical test showed that data point was outlier this point was excluded.
Reproducibility	NPQ kinetics results were successfully repeated in two independent field locations. The quantification ofxanthophyll pigments, ascorbate and dehydroascorbate were extended to additional two genotypes to show successfully that the same results can be obtained in different genotypes with similar chilling tolerance. The hyperspectral indices for which we relied on natural occurrence of chilling events we were not able to measure in other time window were warm day and warm night were in close approximation to chilling day and chilling night with all three genotypes heaving still green leaves and morning being bright. We did not repeat the leaf infiltration due to limitation in access to high light growth chamber where plants were grown to this experiment. The gene expression analyses were not repeated by us.
Randomization	Collection of biological replicates of different genotypes was randomized inside the set of three plants each representing one of

Randomization | three investigated genotypes (or set of five in the case of experiments with 5 lines). For the growth chamber experiment, pots were randomized and placed in a growth chamber. For field, a completely randomized block design with four blocks was established.

Blinding | We do not have qualitative data in our study which collection could be affected by the objectiveness of the researcher. We used the software to detect the data and statistical programs to analyze our quantitative data.

Did the study involve field work? ☒ Yes ☐ No

Field work, collection and transport

Field conditions	The UIUC field experiment (40.067 N, 88.198 W) was planted on drummer silty clay loam on April 29-30, 2015. Single-plant plots were established at a spacing of 2.0 m x 2.0 m in a completely randomized block design with four blocks of 324 plots in an 18 x 72 grid and fertilized each spring with nitrogen fertilizer (80 kg ha-1). In each block, 321 F2 progenies derived from a cross between MsiCR and MsaRB were planted, together with both parents and Mxgl added as a chilling-tolerant control. The collection of leaf discs was performed in summer 2017 from MsaRB, Mxgl and MsiCR plants. The F2 population was not part of this study. The field experiment at the University of Nebraska-Lincoln (UNL; 40.829 N, 96.657 W) was planted on silt clay loam soil on June 7, 2019. A completely randomized block design with four blocks was established. Each block contained four single-plant plots of each of the three Miscanthus accessions: MsaRB, Mxgl and MsiCR. The plots were organized in a 6 x 8 grid with 1.5-m spacing between plots. For both field trials, experimental plots were surrounded by a single row of Mxgl; weeds were controlled mechanically and/or with herbicides as needed. Climate data were recorded at weather stations near the field trial locations and are available as supplemental figure.
Location	University of Illinois Urbana-Champaign field experiment (UIUC; 40.067 N, 88.198 W); University of Nebraska-Lincoln (UNL; 40.829 N, 96.657 W)
Access & import/export	The interventions at the fields were minimized to one herbicide application and cutting yearly the biomass in late Spring. Plants at UNL were cut manually and mechanically in the bigger field at UIUC. No permits are required to grow Miscanthus in the US. Both locations offields experiments are university properties.
Disturbance	Planting and most of the field management was done manually.

Reporting for specific materials, systems and methods

We require information from authors about some types of materials, experimental systems and methods used in many studies. Here, indicate whether each material, system or method listed is relevant to your study. If you are not sure if a list item applies to your research, read the appropriate section before selecting a response.

Materials & experimental systems

n/a	Involved in the study	
☒	☐	Antibodies
☒	☐	Eukaryotic cell lines
☒	☐	Palaeontology and archaeology
☒	☐	Animals and other organisms
☒	☐	Clinical data
☒	☐	Dual use research of concern
☐	☒	Plants

Methods

n/a	Involved in the study	
☒	☐	ChIP-seq
☒	☐	Flow cytometry
☒	☐	MRI-based neuroimaging

Dual use research of concern

Policy information about dual use research of concern

Hazards

Could the accidental, deliberate or reckless misuse of agents or technologies generated in the work, or the application of information presented in the manuscript, pose a threat to:

No	Yes	
☒	☐	Public health
☒	☐	National security
☒	☐	Crops and/or livestock
☒	☐	Ecosystems
☒	☐	Any other significant area

Experiments of concern

Does the work involve any of these experiments of concern:

No	Yes	
☒	☐	Demonstrate how to render a vaccine ineffective
☒	☐	Confer resistance to therapeutically useful antibiotics or antiviral agents
☒	☐	Enhance the virulence of a pathogen or render a nonpathogen virulent
☒	☐	Increase transmissibility of a pathogen
☒	☐	Alter the host range of a pathogen
☒	☐	Enable evasion of diagnostic/detection modalities
☒	☐	Enable the weaponization of a biological agent or toxin
☒	☐	Any other potentially harmful combination of experiments and agents

Plants

Seed stocks	Five accessions were chosen from the Miscanthus diversity collection of the University of Illinois Urbana-Champaign (UIUC) to represent a broad gradient of chilling tolerance. Two M. sacchariflorus accessions, Robustus-Blumel (MsaRB; Ull0-00009) and 'RU2012-114' (MsaRU; RU2012-114) show exceptionally high chilling tolerance. Two M. xgiganteus accessions, 'Illinois' (Mxgl) (UI10-00107) and 'Nagara' (MxgN; UI10-00123), have moderate chilling tolerance. M. sinensis var. condensatus 'Cosmo Revert' (MsiCR; UI10-00014) has low chilling tolerance. All rhizomes were multiplied vegetatively in the greenhouse.
Novel plant genotypes	N/A
Authentication	The material was obtained from the Miscanthus breeder at UIUC as a rhizome pieces and clonally propagated. The genotypes used in this study are easily phenotypically distinguishable.
